# Was a Quarter of a Grain Too Large a Dose?

**Published:** 1877-11

**Authors:** 


### WAS A QUARTER OF A GRAIN TOO LARGE A DOSE?

In the August (1877) number of the Chicago Medical Journal and Examiner, Dr. Ingals published a paper, entitled: "Danger from Hypodermic Injection," in which the doctor says: "I was called in the night to see a woman, who, from continuous watching with sick children, had become debilitated, and, as a result, suffered at times from severe pains of a neuralgic character. * * * I dissolved one-fourth of a grain of morphia in pure water, and administered it under the integuments on the outer side of the arm.

The doctor then states, "in a few seconds the breathing becomes stertorous; the pulse failed; the lips and countenance become livid, etc., etc." The woman, he tells us, was, however, resuscitated.

The paper also contained reports of cases Nos. 1, 2 and 3, by E. Wenger, M. D. Cases 1 and 3 died; but the doctor did not state the dose administered in either case. Now this article may have one of two effects upon the profession; first, it may deter physicians from using the hypodermic syringe in cases where it would be the means of saving life; and, secondly, it may induce others to use morphia hypodermically in doses large enough to destroy life. I have used morphia hypodermically for the last ten years, and have not had a single bad result, other than an occasional inflammation at the point of introduction. My rule has been to always commence with a dose one-third the amount I would have given internally, and never without atropia.

Prof. R. Bartholow, in his excellent work on Hypodermic Medication, says, on page 41: "The dose of morphia for hypodermic injections varies from 1/[illegible] to $\frac{1}{2}$ of a grain. In commencing it should not exceed *one-third* of that ordinarily administered internally. It is prudent in all cases to test the physiological capabilities by a moderate dose before resorting to the maximum amount. Women are, as a rule, more easily affected than men; $\frac{1}{12}$ of a grain is a sufficient dose for many of the conditions requiring an injection. Maximum doses may be administered with safety if combined with atropia." And on page 102, "atropia in small doses—$\frac{1}{76}$ of a grain—increases the hypnotic power of morphia. The pain-relieving power of morphia is increased by atropia. When toxic doses are used, the narcotism of morphia is overcome by atropia, and vice versa." Prof. H. C. Wood, of Philadelphia, in his work on Materia Medica, page 218, says: "I have seen very alarming results from the injection of one-sixth of a grain, and one-half a grain has produced death. In females, unless very robust, the maximum dose should be $\frac{1}{8}$ of a grain; in men one-sixth to one-quarter."

With the evidence given, and the effects of one-fourth of a grain upon the doctor's patient, the reader will be able to answer the question that heads this paper.

Du Quoin, Ill. JOHN MCLEAN.

For the information of the reader who desires to answer the foregoing questions, I may state that, in the case which I reported, the dose was not large enough to relieve the pain for any considerable time, and it was not sufficient to cause sleep.

The writer fears that the report will have the effect, either to encourage the use of the medicine in excessive doses, or to deter physicians from administering it by the hypodermic method when it may be needed.

Certainly the unfortunate result from one-fourth of a grain cannot induce physicians to give larger quantities; and as to the second criticism—if the report induces greater caution in the administration of remedies, it will have accomplished the end for which it was intended.

Regarding the usual dose of morphia, and the dangers from hypodermic injections, I will have something to say in a future number. E. FLETCHER INGALS.

